# Supplementary material for: Diversity, Prevalence and Virulence of Colletotrichum Species Causing Anthracnose on Cassava Leaves in the Northern Region of Brazil
Source: J Fungi (Basel). 2024 May 21;10(6):367. doi: 10.3390/jof10060367 (PMC11205099; doi:10.3390/jof10060367)
Supplement: Supplementary file 1 [file jof-10-00367-s001.zip › jof-2988555-supplementary.pdf]

Table S1. Primers used in this study (sequence and reference)

Gene	Primer	Sequence (5′–3′)	Reference
ACT	ACT-512F	ATGTGCAAGGCCGGTTTCGC	(Carbone and Kohn, 1999)
	ACT-783R	TACGAGTCCTTCTGGCCCAT	
APN2/MAT-GS	CgDL_F6	AGTGGAGGTGCGGGACGTT	(Rojas et al., 2010)
	CgMAT1_F2	TGATGTATCCCGACTACCG	
APN2	CgDL_R1	GCCCGACGAGCAGAGGACGTAGTC	(Rojas et al., 2010)
	ColDL_F3	GGGAGAAGCGAACATACCA	
CAL	CL1C	GAA TTC AAG GAG GCC TTC TC	(Weir et al., 2012)
CHS-1	CL2C	CTT CTG CAT CAT GAG CTG GAC	
	CHS-354R	TGGAAGAACCATCTGTGAGAGTTG	(Carbone and Kohn, 1999)
	CHS-79F	TGGGGCAAGGATGCTTGGAAGAAG	
GAPDH	GAP-95	CCGTCAACGACCCCTTCATT	(Vieira et al., 2017)
	GAP-1174	AACCCCACTCGTTGTCGTAC	
GAP2-IGS	GAP-1041	CTACACCGAGGACGATGTCG	(Vieira et al., 2017)
	GAP/IGS-2044	TTCTACGGGAAAACCAGGGC	
HIS3	CYLH3F	AGG TCC ACT GGT GGC AAG	(Crous et al., 2004)
	CYLH3R	AGC TGG ATG TCCTTG GAC TG	
TUB2	BT1	AACATGCGTGAGATTGTAAGT	(O'Donnell and Cigelnik, 1997)
	BT22	TCTGGATGTTGTTGGGAATCC	

Source Universidade Federal Rural de Pernambuco, 2020.

Supplementary table S2. *Colletotrichum* species complex, strains of *Colletotrichum* spp. included in the present study and GenBank accession numbers.

***Colletotrichum* species complex**	***Colletotrichum* species**	**Culture number***	**GenBank accession number**								
			**ACT**	**APN2**	**APN2/MAT -IGS**	**CAL**	**CHS-1**	**GAP2-IGS**	**GAPDH**	**HIS3**	**TUB2**
Boninense	*C. annellatum*	CBS 129826*	-	-	-	JQ005743	-	-	JQ005309	-	JQ005656
Boninense	*C. beeveri*	CBS 128527*	-	-	-	JQ005692	-	-	JQ005258	-	JQ005605
Boninense	*C. boninense*	CBS 123755*	-	-	-	JQ005674	-	-	JQ005240	-	JQ005588
Boninense	*C. brasiliense*	CBS 128528*	-	-	-	JQ005755	-	-	JQ005321	-	JQ005668
Boninense	*C. brassicicola*	CBS 101059*	-	-	-	JQ005693	-	-	JQ005259	-	JQ005606
Boninense	*C. catinaense*	CBS 142417*	-	-	-	KY856053	-	-	KY856224	-	KY856482
Boninense	*C. colombiense*	CBS 129818*	-	-	-	JQ005695	-	-	JQ005261	-	JQ005608
Boninense	*C. constrictum*	CBS 128504*	-	-	-	JQ005759	-	-	JQ005325	-	JQ005672
Boninense	*C. cymbidiicola*	IMI 347923*	-	-	-	JQ005687	-	-	JQ005253	-	JQ005600
Boninense	*C. dacrycarpi*	CBS 130241*	-	-	-	JQ005757	-	-	JQ005323	-	JQ005670
Boninense	*C. hippeastri*	CBS 125376*	-	-	-	JQ005752	-	-	JQ005318	-	JQ005665
Boninense	*C. karsti*	CBS 110779	-	-	-	JQ005730	-	-	JQ005296	-	JQ005643
Boninense	*C. karsti*	CBS 127597	-	-	-	JQ005725	-	-	JQ005291	-	JQ005638
Boninense	*C. karsti*	CBS 111998	-	-	-	JQ005733	-	-	JQ005299	-	JQ005646
Boninense	*C. karsti*	CBS 112762	-	-	-	JQ005734	-	-	JQ005300	-	JQ005647
Boninense	*C. karsti*	CBS 126532	-	-	-	JQ005730	-	-	JQ005296	-	JQ005643
Boninense	*C. karsti*	**UFT/Coll50.1**				MT396233			MT800858		MT856674
Boninense	*C. karsti*	**UFT/Coll61**				MT396234			MT800859		MT856675
Boninense	*C. limonicola*	CBS 142410*	-	-		KY856125	-	-	KY856296	-	KY856554
Boninense	*C. novae-zelandiae*	CBS 128505*	-	-		JQ005749	-	-	JQ005315	-	JQ005662
Boninense	*C. oncidii*	CBS 129828*	-	-		JQ005690	-	-	JQ005256	-	JQ005603
Boninense	*C. parsonsiae*	CBS 128525*	-	-		JQ005754	-	-	JQ005320	-	JQ005667

Boninense	*C. petchii*	CBS 378.94*	-	-		JQ005744	-	-	JQ005310	-	JQ005657
Boninense	*C. phyllanthi*	CBS 175.67*	-	-		JQ005742	-	-	JQ005308	-	JQ005655
Boninense	*C. torulosum*	CBS 128544*	-	-		JQ005685	-	-	JQ005251	-	JQ005598
Boninense	*C. camelliae-japonicae*	CGMCC3.18118 *	-	-		KC293696	-	-	KX893584	-	KX893580
Boninense	*C. citricola*	CBS 134228*	-	-		KC293697	-	-	KC293736	-	KC293656
Gloeosporioides	*C. aenigma*	ICMP 18608*	-	-	KM360143	-	-	-	-	-	-
Gloeosporioides	*C. aeschynomenes*	ICMP 17673*	-	-	KM360145	-	-	-	-	-	-
Gloeosporioides	*C. alienum*	ICMP 12071*	-	-	KC888927	-	-	-	-	-	-
Gloeosporioides	*C. asianum*	ICMP 18580*	-	-	FR718814	-	-	-	-	-	-
Gloeosporioides	*C. chrysophilum*	CMM4268*	-	KX094018	KX094325	KX094063	-	KX094125	KX094183	-	KX094285
Gloeosporioides	*C. chrysophilum*	CMM4394	-	KX094006	KX094321		-	KX094129	-	-	-
Gloeosporioides	*C. chrysophilum*	Coll919	-	JX145265	JX145317	KX094057	-	KX094127	KX094177	-	KX094288
Gloeosporioides	*C. chrysophilum*	**UFT/Coll69**		MT396231	MT409462		MT759633				
Gloeosporioides	*C. chrysophilum*	**UFT/Coll82**		MT396232	MT396239		MT800846				
Gloeosporioides	*C. fragariae*	CBS 142.31*	-	-	JQ807844	-					
Gloeosporioides	*C. fructicola*	3589	-	GU994411	GU994440	-		KX094122	-	-	-
Gloeosporioides	*C. fructicola*	CBS 125397	-	GU994412	JQ807839	-		-	-	-	-
Gloeosporioides	*C. fructicola*	Coll1126	-	JX145239	JX145315	-		-	-	-	-
Gloeosporioides	*C. fructicola*	GM567= MTCC 11679	-	-	JQ894576	-		-	-	-	-
Gloeosporioides	*C. fructicola*	**UFT/Coll64**		MT800865	MT396240			MT800847			
Gloeosporioides	*C. fructicola*	**UFT/Coll83**		MT800866	MT409463			MT800848			
Gloeosporioides	*C. gloeosporioides*	IMI 356878*	-	GU994416	JQ807843	-	-	-	-	-	-
Gloeosporioides	*C. horii*	ICMP 10492*	-		JQ807840	-	-	-	-	-	-
Gloeosporioides	*C. musae*	CBS 116870*	-	-	KC888926	-	-	-	-	-	-
Gloeosporioides	*C. nupharicola*	CBS 470.96*	-	JX145275	JX145319	-	-	-	-	-	-
Gloeosporioides	*C. perseae*	CBS141365*	-		KX620177	-	-	-	-	-	-
Gloeosporioides	*C. queenslandicum*	ICMP 1778*	-	-	KC888928	-	-	-	-	-	-
Gloeosporioides	*C. salsolae*	ICMP 19051*	-	-	KC888925	-	-	-	-	-	-
Gloeosporioides	*C. siamense*	CMM4244	-	KX094014	KX094315	KX094055	-	KX094135	KX094172	-	KX094299

Gloeosporioides	*C. siamense*	CMM4247	-	KX094009	KX094301	KX094038	-	KX094141	KX094155	-	KX094261
Gloeosporioides	*C. siamense*	CMM4248	-	KX093992	KX094314	KX094037	-	KX094136	KX094154	-	KX094300
Gloeosporioides	*C. siamense*	CMM 3777	-	KX093993	KX094302	KX094045	-	KX094142	KX094162	-	KX094266
Gloeosporioides	*C. siamense*	CBS133123	-	JX145245	JX145309	KX094035	-	KX094133	KX094186	-	KX094289
Gloeosporioides	*C. siamense*	ICMP 18578*	-	-	JQ899289	-	-	-	-	-	-
Gloeosporioides	*C. siamense*	**UFT/Coll44**		MT396236	MT800867			MT800849			
Gloeosporioides	*C. siamense*	**UFT/Coll51**		MT396237	MT800868			MT800850			
Gloeosporioides	*C. siamense*	**UFT/Coll63**		MT396238	MT759634			MT800851			
Gloeosporioides	*C. tainanense*	CBS 143666	-	-	MH728836	-	-	-	-	-	-
Gloeosporioides	*C. theobromicola*	CBS 124945	-	GU994419	KC790726	-	-	-	-	-	-
Gloeosporioides	*C. tropicale*	CBS 124949*	-	GU994396	GU994425	-	-	-	-	-	-
Orchidearum	*C. cattleyicola*	CBS 170.49*	-	-	-	-	MG600866	-	MG600819	MG600905	MG601025
Orchidearum	*C. clivicola*	CBS 125375*	-	-	-	-	MG600850	-	MG600795	MG600892	MG601000
Orchidearum	*C. musicola*	CBS 132885*	-	-	-	-	MG600853	-	MG600798	MG600895	MG601003
Orchidearum	*C. musicola*	CBS 127557	-	-	-	-	MG600854	-	MG600799	MG600896	MG601004
Orchidearum	*C. musicola*	**UFT/Coll89**					MT396235		MT800856	MT800870	MT856672
Orchidearum	*C. orchidearum*	CBS 135131*	-	-	-	-	MG600855	-	MG600800	MG600897	MG601005
Orchidearum	*C. piperis*	CPC 21195*	-	-	-	-	MG600867	-	MG600820	MG600906	MG601027
Orchidearum	*C. plurivorum*	CBS 125474*	-	-	-	-	MG600841	-	MG600781	MG600887	MG600985
Orchidearum	*C. plurivorum*	CBS 125473	-	-	-	-	MG600840	-	MG600780	MG600886	MG600984
Orchidearum	*C. plurivorum*	CBS 132443	-	-	-	-	MG600842	-	MG600782	MG600888	MG600986
Orchidearum	*C. plurivorum*	***UFT/Coll23***					MT800869		MT800852	MT800861	MT856985
Orchidearum	*C. plurivorum*	**UFT/Coll31**					MT800870		MT800853	MT800862	MT856986
Orchidearum	*C. plurivorum*	**UFT/Coll70**					MT800872		MT800855	MT800864	MT856988
Orchidearum	*C. plurivorum*	***UFT/Coll67***					MT800871		MT800854	MT800863	MT856987
Orchidearum	*C. sojae*	ATCC 62257*	-	-	-	-	MG600860	-	MG600810	MG600899	MG601016

Orchidearum	*C. vittalense*	CBS 181.82*	-	-	-	-	MG600851	-	MG600796	MG600893	MG601001
Truncatum	*C. acidae*	MFLUCC 172659*	MH003697	-	-	-	-	-	MH003691	-	MH003700
Truncatum	*C. curcucmae*	IMI 288937*	GU227991	-	-	-	-	-	GU228285	-	GU228187
Truncatum	*C. fusiforme*	MFLUCC 120437*	KT290251	-	-	-	-	-	KT290255	-	KT290256
Truncatum	*C. truncatum*	CBS 151.35*	GU227960	-	-	-	-	-	GU228254	-	GU228156
Truncatum	*C. truncatum*	CBS 195.32	GU227963	-	-	-	-	-	GU228257	-	GU228159
Truncatum	*C. truncatum*	CBS 182.52	GU227964	-	-	-	-	-	GU228258	-	GU228160
Truncatum	*C. truncatum*	CAUT30	KP145501	-	-	-	-	-	KP145569	-	0
Truncatum	*C. truncatum*	**UFT/Coll87**	MT321653						MT800857		MT856675

. CBS = Centraalbureau voor Schimmelcultures (Netherlands), ICMP = International Collection of Microorganisms from Plants, MFLUCC = Mae Fah Luang University, IMI = CABI Genetic Resource Collection (UK),CMM = Culture Collection of Phytopathogenic Fungi “Prof. Maria Menezes”, Universidade Federal Rural de Pernambuco (Brazil), CGMCC: China General Microbiological Culture Collection; MTCC: Microbial type culture collection and gene bank, India. "" indicates ex-holotype or ex-epitype cultures. 2. Sequences generated in the present study are emphasized in **bold**. "-"indicates missing data.

Source: Universidade Federal Rural de Pernambuco, 2020
